# Antinociceptive and Antioxidant Activity of *Zanthoxylum budrunga* Wall (Rutaceae) Seeds

**DOI:** 10.1155/2014/869537

**Published:** 2014-02-23

**Authors:** Md. Khirul Islam, Nripendra Nath Biswas, Sanjib Saha, Hemayet Hossain, Ismet Ara Jahan, Tanzir Ahmed Khan, Khalijah Awang, Jamil A. Shilpi

**Affiliations:** ^1^Pharmacy Discipline, Life Science School, Khulna University, Khulna 9208, Bangladesh; ^2^School of Chemistry, University of New South Wales, Sydney, NSW 2052, Australia; ^3^Chemical Research Division, BCSIR Laboratories, Bangladesh Council of Scientific and Industrial Research (BCSIR), Dhaka 1205, Bangladesh; ^4^Department of Chemistry, Faculty of Science, University of Malaya, 50603 Kuala Lumpur, Malaysia; ^5^Centre for Natural Products and Drug, University of Malaya, 50603 Kuala Lumpur, Malaysia

## Abstract

Different parts of the medicinal plant *Zanthoxylum budrunga* Wall enjoy a variety of uses in ethnobotanical practice in Bangladesh. In the present study, a number of phytochemical and pharmacological investigations were done on the ethanol extract of *Z. budrunga* seeds (ZBSE) to evaluate its antinociceptive and antioxidant potential. ZBSE was also subjected to HPLC analysis to detect the presence of some common antioxidants. In acetic acid induced writhing test in mice, ZBSE showed 65.28 and 74.30% inhibition of writhing at the doses of 250 and 500 mg/kg and the results were statistically significant (*P* < 0.001). In hot-plate test, ZBSE raised the pain threshold significantly (*P* < 0.001) throughout the entire observation period. In DPPH scavenging assay, the IC_50_ of ZBSE was observed at 82.60 *μ*g/mL. The phenolic content was found to be 338.77 mg GAE/100 g of dried plant material. In reducing power assay, ZBSE showed a concentration dependent reducing ability. HPLC analysis indicated the presence of caffeic acid with a concentration of 75.45 mg/100 g ZBSE. Present investigation supported the use of *Zanthoxylum budrunga* seed in traditional medicine for pain management. Constituents including caffeic acid and other phenolics might have some role in the observed activity.

## 1. Introduction

*Zanthoxylum budrunga *Wall (Syn: *Zanthoxylum rhetsa *Roxb.) (Rutaceae) is a deciduous medium-sized tree native to subtropical areas of the world and widely distributed in Bangladesh, India, Sri Lanka, Myanmar, China, Thailand, Malaysia, Indonesia, Philippines, and Papua New Guinea. It grows up to 35 m with sparsely armed branches having straight or ascending prickles. In Bangladesh, it is distributed in Sylhet, Chittagong Hill Tracts, Cox's Bazar, Gazipur, and Tangail and enjoys a number of uses in ethnobotanical practice. In traditional medicine, the fruit of the plant is used in the treatment of rheumatism, cholera, dysentery, asthma, infections, heart disease, bronchitis, and piles. The essential oil extracted from leaves is used in cholera. Juice of the bark is considered beneficial in vomiting, cough, dysentery, and headache [1]. The seed oil is antiseptic and is used in the treatment of inflammatory dermatitis, dry eczema, and dandruff [2]. In a previous study, the methanol extract of stem bark showed antinociceptive and antidiarrhoeal activity in experimental mice [3]. In another study, three terpenes isolated from the bark showed cytotoxic activity, although no names or structures of the compounds were available in the report [4]. Several alkaloids and lignans were isolated from the stem bark [5]. The chemical composition of the volatile oil of seed coat was analyzed and thirty-four compounds were identified including terpinen-4-ol (32.1%), *α*-terpineol (8.2%), sabinene (8.1%), *β*-phellandrene (7.4%), and 2-undecanone (7.1%) [6]. Volatile constituents (hydroxy *α*-sanshool, *β*-phellandrene, *β*-pinene and pipertone, mullilam diol) and alkaloids (glycosine, rutaecarpine) were isolated from the fruits [7–9]. A monoterpene triol, trihydroxy-*p*-menthane, was reported from the roots, [10] while three alkaloids, namely, pseudophrynamine, 2-(2′,4′,6′-trimethyl heptenyl)-4-quinozolone, and lunacridine, were isolated from the leaves [11]. The present study was designed to evaluate antinociceptive and antioxidant activities of the ethanol extract of *Z. budrunga* seeds.

## 2. Materials and Methods

### 2.1. Plant Material

The seeds of *Z. budrunga* was collected from Tangail, Bangladesh, in September 2011 and identified by experts at Bangladesh National Herbarium, Dhaka, Bangladesh. A voucher specimen (DACB 37522) has been submitted there for future reference. The seeds were removed from the fruit rind and separated from other parts and extraneous materials.

### 2.2. Extraction

The dried seeds were grinded into coarse powder (210 g) and macerated in ethanol (95%, 1 L) for three days with occasional shaking. It was filtered through a clear cotton plug to remove marc. Filtrate was evaporated to get gummy reddish crude extract. The seeds yielded 1.45% extract of dried plant material and designated as *Z. budrunga* seed extract (ZBSE).

### 2.3. Test Animals

Randomly screened young Swiss-Albino mice aged 4-5 weeks, weighing 20–25 g, were selected for the pharmacological investigation. The mice were purchased from the Animal Resources Branch of International Centre for Diarrhoeal Disease and Research, Bangladesh (ICCDR,B), and fed with ICCDR,B formulated commercial rodent food and water *ad libitum*. The investigations were carried out following the animal ethics guidelines provided by ICDDR,B.

### 2.4. Chemicals and Reagents

Gallic acid, (+)-catechin hydrate, vanillic acid, caffeic acid, (−)-epicatechin, *p*-coumaric acid, rutin hydrate, ellagic acid, quercetin, and 1,1-Diphenyl-2-picrylhydrazyl (DPPH) were purchased from Sigma-Aldrich, USA. Folin-Ciocalteu's reagent, ascorbic acid, trichloroacetic acid, potassium ferricyanide, sodium carbonate, ferric chloride, acetic acid, and dimethyl sulfoxide (DMSO) were purchased from Merck, Germany. Tween-80 was purchased from Loba Chemie, India. Diclofenac sodium, morphine, and vincristine sulphate were obtained from Beximco Pharmaceuticals Ltd Bangladesh, Popular Pharmaceuticals Ltd Bangladesh, and Cipla Pharmaceuticals India, respectively.

### 2.5. Phytochemical Analysis

ZBSE was subjected to a number of standard tests to detect the presence of major phytochemical groups [12, 13].

### 2.6. DPPH Radical Scavenging Activity

*In vitro *antioxidant activity was determined based on the ability of the seed extract to scavenge the stable free radical DPPH [14]. Stock solution of ZBSE was prepared in ethanol and serially diluted to obtain different concentrations with the maximum and minimum of 512 and 1 *μ*g/mL, respectively. From each concentration, 1 mL was mixed with 2 mL DPPH solution (0.004% in ethanol). The mixture was kept for 30 minutes to complete the reaction. The absorbance was measured at 517 nm in a UV-visible spectrophotometer. Ascorbic acid was used as standard antioxidant. The percent inhibition was calculated according to the formula (1 − *A*_1_/*A*_0_) × 100, where *A*_0_ is the absorbance of control and *A*_1_ is the absorbance of ZBSE or standard. IC_50_ value was determined from the graph obtained by plotting % inhibition versus concentration.

### 2.7. Determination of Total Phenolic Content (TPC)

Grinded plant material (0.5 g) was mixed with 50 mL of 80% aqueous methanol and sonicated for 20 min. An aliquot of 2 mL was taken from it and centrifuged for 15 min at 14,000 rpm. Total phenolic content was determined by Folin-Ciocalteu's reagent [15]. Standard gallic acid solutions were prepared by serial dilution to get concentrations of 500, 250, 125, 62.5, 31.25, and 15.62 mg/L. Gallic acid solutions of each concentration and ZBSE of 1 mL were transferred to 25 mL volumetric flasks and 9 mL distilled water was added. Folin-Ciocalteu's reagent of 1 mL was added to each volumetric flask with continuous shaking. After 5 min, 10 mL of 7% Na_2_CO_3_ was added and adjusted with distilled water to make the final volume of 25 mL. It was kept for 30 min to complete any reaction that occurs. The absorbance was measured at 750 nm against blank. Blank was prepared by following the same steps mentioned above without adding ZBSE or gallic acid. Standard curve of gallic acid was prepared by plotting absorbance versus concentration. The total phenolic content of ZBSE was determined using the standard calibration curve of gallic acid and the value was expressed as gallic acid equivalent (GAE)/100 g of dried plant material.

### 2.8. Reducing Power Assay

Reducing power of the extract was determined according to the method described by Oyaizu [16]. ZBSE of 1 mL, with the concentrations of 500, 250, 125, 62.5, 31.25, and 15.62 *μ*g/mL were mixed with 2.5 mL of 0.2 M phosphate buffer (pH 6.6) and 2.5 mL of 1% potassium ferricyanide with vigorous shaking. The mixtures were incubated for 20 min at 50°C. After cooling at room temperature, 2.5 mL of 10% trichloroacetic acid was added and centrifuged at 3000 rpm for 10 min. An aliquot (2.5 mL) of the supernatant was mixed with 2.5 mL of distilled water and 0.5 mL of ferric chloride (0.1%). After 5 min, the absorbance was measured at 700 nm. Ascorbic acid was used as standard.

### 2.9. HPLC Detection of Phenolics

Detection of selected phenolic compounds in ZBSE was determined by HPLC analysis as described by Chuanphongpanich and Phanichphant with some modifications [17]. It was carried out on a Dionex UltiMate 3000 system equipped with quaternary rapid separation pump (LPG-3400RS) and photodiode array detector (DAD-3000RS). Separation was performed using Acclaim C_18_ (5 *μ*m) Dionex column (4.6 × 250 mm) at 30°C with a flow rate of 1 mL/min and an injection volume of 20 *μ*L. The mobile phase consisted of acetonitrile (solvent A), acetic acid solution pH 3.0 (solvent B), and methanol (solvent C) with the gradient elution program of 5%A/95%B (0–9 min), 10%A/80%B/10%C (10–19 min), and 20%A/60%B/20%C (20–30 min) with postrun equilibration of the system with 100%A (5 min). The UV detector was set to 280 nm for 18.0 min, changed to 320 nm for 6 min and finally to 380 nm, and held for the rest of the analysis period while the diode array detector was set at an acquisition range from 200 nm to 700 nm. For the preparation of calibration curve, a standard stock solution was prepared in methanol containing gallic acid, vanillic acid, (+)-catechin, (−)-epicatechin, *p*-coumaric acid, rutin, ellagic acid (20 *μ*g/mL each), caffeic acid (8 *μ*g/mL), and quercetin (6 *μ*g/mL). A solution of ZBSE was prepared in methanol having a concentration of 5 mg/mL. Prior to HPLC analysis, all solutions (mixed standards, sample, and spiked solutions) were filtered through 0.2 *μ*m nylon syringe filter (Sartorius, Germany) and degassed in an ultrasonic bath (Hwashin, Korea) for 15 min. Data acquisition, peak integration, and calibrations were performed with Dionex Chromeleon software (Version 6.80 RS 10).

### 2.10. Acetic Acid Induced Writhing Test

Antinociceptive activity was evaluated by acetic acid induced writhing model in mice [18]. Control group received vehicle (1% tween-80 in distilled water) at the dose of 10 mL/kg. Positive control group was treated with diclofenac sodium at the dose of 25 mg/kg body weight and test groups with ZBSE at the doses of 250 and 500 mg/kg body weight. All the treatments were administered orally. For proper absorption of the extract or drug, a period of 30 min was provided followed by the intraperitoneal administration of acetic acid (0.7%) at the dose of 10 mL/kg to induce abdominal contraction or writhing. After an interval of 5 min, writhing was counted for 10 min. Total number of writhing of positive control group and test groups was compared with the control group.

### 2.11. Hot-Plate Test

In hot-plate test, experimental mice were subjected to pain stimulus when placed on hot-plate maintained at the temperature of 55 ± 0.5°C [19]. Mice, having latency time of 3 to 5 sec, were selected for the study. The latency time was considered as the time between placing the mice on hot plate and the time when mice licked their fore and hind paws or jumped from the plate. Latency time was recorded at 0, 30, 60, 90, and 120 min following the oral administration of vehicle (1% tween-80 in distilled water, 10 mL/kg) for control, morphine (5 mg/kg) for the positive control, and ZBSE (250 and 500 mg/kg) for the test groups. A cut-off point of 15 sec was used to avoid accidental paw damage. Latency time of positive control and test groups was compared with control group.

### 2.12. Statistical Analysis

All the values were expressed as mean ± SEM. Statistical analysis was performed by Student's *t*-test. Experimental results were considered statistically significant when *P* < 0.05.

## 3. Results

### 3.1. Phytochemical Group Test

Phytochemical screening of ZBSE revealed the presence of reducing sugars, alkaloids, glycosides, flavonoids, and gums while saponins, tannins, and steroids were absent (Table 1).

### 3.2. DPPH Scavenging Activity

**Figure 1:**
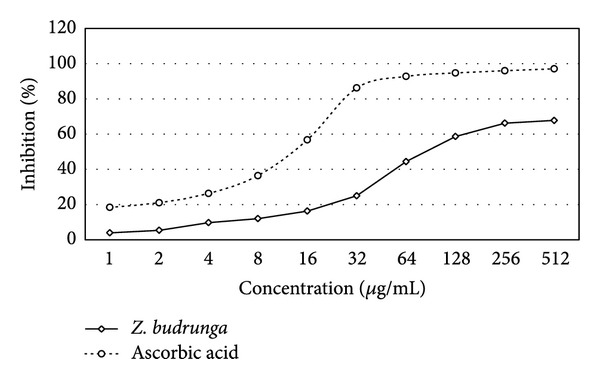
DPPH scavenging activity of *Z. budrunga* seed extract.

ZBSE showed DPPH radical scavenging activity in a concentration dependent manner. The IC_50_ value for the extract was 82.60 *μ*g/mL while the value was 12.58 *μ*g/mL for ascorbic acid, used as standard in this assay (Figure 1).

### 3.3. Phenolic Content Determination

**Figure 2:**
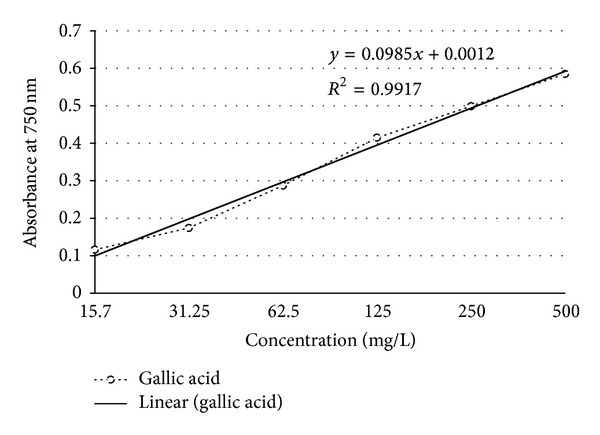
Standard calibration curve of gallic acid for determination of total phenolic content.

Phenolic content of ZBSE was found to be 338.77 mg GAE/100 g of dried plant material. Gallic acid standard calibration curve equation was *y* = 0.098*x* + 0.001, *R* ^2^ = 0.991; where *y* is the absorbance and *x* is the phenolic content expressed in gallic acid equivalent (Figure 2).

### 3.4. Reducing Power Assay

**Figure 3:**
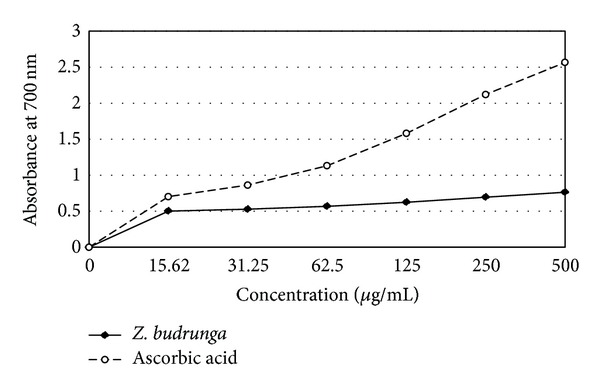
Reducing power of *Z. budrunga* seed extract.

Reducing ability of ZBSE increased in a concentration dependent manner. At the concentrations of 15.62, 31.25, 62.5, 125, 250, and 500 *μ*g/mL, ZBSE showed absorbances of 0.502, 0.528, 0.568, 0.625, 0.695, and 0.764 while ascorbic acid showed absorbances of 0.702, 0.863, 1.132, 1.581, 2.121, and 2.567, respectively (Figure 3).

### 3.5. HPLC Analysis

**Figure 4:**
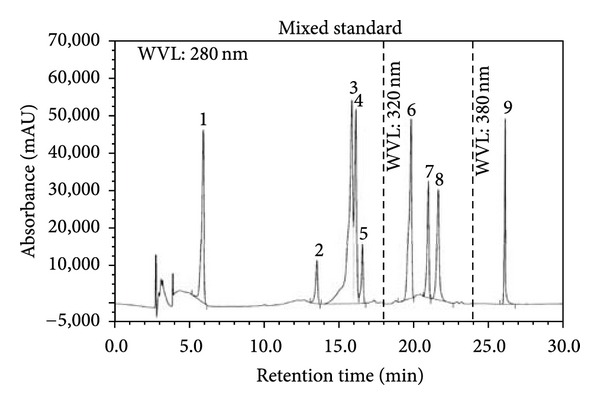
HPLC chromatogram of a standard mixture of polyphenolic compounds (1: gallic acid, 2: (+)-catechin, 3: vanillic acid, 4: caffeic acid, 5: (−)-epicatechin, 6: *p*-coumaric acid, 7: rutin, 8: ellagic acid, and 9: quercetin).

**Figure 5:**
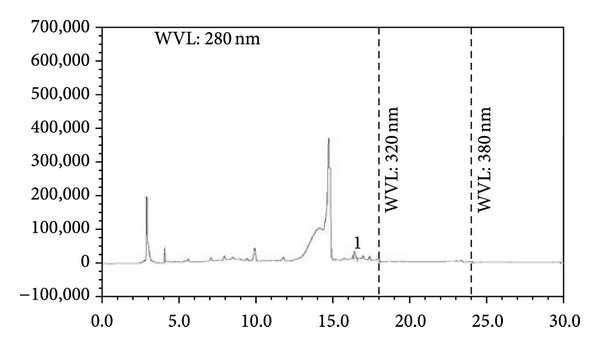
HPLC chromatogram of *Z. budrunga* seed extract (1: caffeic acid).

Among the selected phenolic compounds, only caffeic acid was found to be present in ZBSE with a concentration of 75.45 mg/100 g ZBSE. Gallic acid, vanillic acid, (+)-catechin, (−)-epicatechin, caffeic acid, *p*-coumaric acid, rutin, quercetin, and ellagic acid were absent or were present beyond detection level. The chromatogram also showed peaks in regions that represent simple polyphenols and catechins (Figures 4 and 5 and Tables 2 and 3).

### 3.6. Writhing Test

In acetic acid induced writhing, ZBSE showed 65.28 and 74.30% inhibition of writhing at the doses of 250 and 500 mg/kg body weight, respectively, whereas diclofenac sodium (25 mg/kg) showed 81.95% inhibition (Table 4).

### 3.7. Hot-Plate Test

In hot-plate test, ZBSE increased the latency time to pain stimulus. The maximum latency time for the extract at the doses of 250 and 500 mg/kg body weight was 5.90 ± 0.09 and 6.61 ± 0.07 sec, respectively, whereas morphine (3 mg/kg) showed a maximum latency time of 9.41 ± 0.16 sec (Table 5).

## 4. Discussion

Phytochemicals belonging to the class of alkaloids, glycosides, and flavonoids are often associated with various bioactivities including antioxidant and antinociceptive activities [20]. Results of the phytochemical group tests indicated the presence of some interesting class of secondary metabolites including alkaloids and flavonoids. DPPH radical scavenging activity revealed that *Z. budrunga* seed extract possesses moderate antioxidant activity. Phenolic compounds are one of the major groups of natural antioxidants that neutralize free radicals by redox reactions [21]. Phenolic content determination by Folin-Ciocalteu's reagent allows the estimation of all phenolic compounds present in a sample [22]. The extract was further subjected to reducing power assay since antioxidant activity is based on the reducing ability [23]. The extract showed a high level of phenolics as well as concentration dependent reducing power. HPLC analysis was carried out to detect selected phenolics that are abundant in plants. Although only caffeic acid was detected in the extract, some peaks were also present in regions indicating that the extract might contain compounds belonging to the class of simple polyphenols and catechins [24].

Acetic acid induced writhing is an easy but reliable method to evaluate peripherally acting antinociceptive activity [18]. Acetic acid triggers the liberation of free fatty acids from the phospholipids of the cell membrane, which further converts into various eicosanoids including prostacyclin (PGI_2_) by the enzyme phospholipase A_2_. Synthesis of prostaglandins including PGI_2_ through cyclooxygenase pathway is one of the major causes of pain sensation [25]. Common NSAIDs, including diclofenac sodium, used as positive control in the present study exert their analgesic effect through the inhibition of cyclooxygenase mediated prostaglandin synthesis. Inhibition of writhing in the test groups indicated that the extract might have antinociceptive effect via inhibition of synthesis and release of prostaglandins and other endogenous pain mediators. Caffeic acid, which is present in the extract, is known to exert anti-inflammatory activity through the inhibition of arachidonic acid synthesis [26]. In addition, caffeic acid also inhibits the gene expression of COX-2 enzyme [27]. Caffeic acid is also reported to have antinociceptive activity [28]. However, the low concentration of caffeic acid in the extract and extent of antinociceptive activity suggests that some other compounds present in the extract also contributed to the observed activity. Inflammatory tissue damages liberate reactive oxygen species during the process of phagocytic action at inflammatory sites [29]. Antioxidants can relieve pain through the prevention of lipid peroxidation, that is, prostaglandin synthesis from phospholipids by neutralising reactive oxygen species [30]. Thus, the antioxidants present in the extract might have some association in the observed activity.

The extract showed pain inhibitory activity in hot-plate test which represents centrally acting algesia by inducing thermal pain stimulus through changes in spinal cord level [31]. Natural products of various chemical classes, including alkaloids and flavonoids, can give analgesics effect through the inhibition of opioid and other receptors that act in the modulation of pain in CNS [32–35].

Present investigation supported the traditional use of *Z. budrunga* seeds in the management of pain and antioxidants present in the seeds might have some role in the observed activity.

## Figures and Tables

**Table 1:** Results of phytochemical group test of *Z. budrunga* seed extract.

Phytochemical group	Result
Reducing sugars	+
Alkaloids	+
Glycosides	+
Steroids	−
Gums	+
Saponins	−
Flavonoids	+
Tannins	−

+: present; −: absent.

**Table 2:** Parameters of calibration graphs for the nine phenolic standards used.

Peak no.	Polyphenolic compound	Linearity range (µg/mL)	Correlation coefficients (*r* ^2^)	Detection limit (µg/mL)^†^	Quantitation limit (µg/mL)^†^	Recovery (%)^‡^
1	Gallic acid	1.25–20	0.9957	0.25	0.85	98.3 ± 2.79
2	(+)-Catechin	1.25–20	0.9966	0.30	1.12	96.7 ± 1.65
3	Vanillic acid	1.25–20	0.9958	0.21	1.01	97.9 ± 2.85
4	Caffeic acid	0.50–8.0	0.9975	0.14	0.47	102.2 ± 3.19
5	(−)-Epicatechin	1.25–20	0.9955	0.35	1.20	98.3 ± 2.88
6	*p*-Coumaric acid	1.25–20	0.9992	0.26	1.02	103.1 ± 2.74
7	Rutin	1.25–20	0.9986	0.28	1.09	102.8 ± 3.20
8	Ellagic acid	1.25–20	0.9990	0.31	1.17	99.2 ± 2.02
9	Quercetin	0.375–6.0	0.9991	0.11	0.37	100.3 ± 3.95

^†^Data were expressed as the mean of triplicate measurements.

^‡^Recoveries expressed as mean ± standard deviation carried out on ZBSE.

**Table 3:** Contents of polyphenolic compounds in *Z. budrunga* seed extract (*n* = 5).

Polyphenolic compound	*Z. budrunga* seeds	
	Content (mg/100 g of dry extract)	% RSD
Caffeic acid	75.45	0.96

**Table 4:** Effect of *Z*. *budrunga* seed extract on acetic acid induced writhing in mice.

Treatment (*n* = 6)	Dose (mg/kg)	Number of writhes	% inhibition
Control	—	28.8 ± 1.16	—
Diclofenac sodium	25	5.2 ± 0.37*	81.95
ZBSE	250	10.2 ± 0.71*	65.28
	500	7.4 ± 0.68*	74.30

Values are expressed as mean ± standard error for mean; *n*: number of mice (6); Student's *t*-test, *P < 0.001 versus control.

**Table 5:** Effect of *Z. budrunga* seed extract in hot-plate test on mice.

Treatment (*n* = 6)	Dose (mg/kg)	Latency time (sec)				
		0 min	30 min	60 min	90 min	120 min
Control	—	3.26 ± 0.04	3.26 ± 0.04	3.27 ± 0.08	3.23 ± 0.07	3.23 ± 0.06
Morphine	5	3.61 ± 0.03*	6.38 ± 0.15*	7.38 ± 0.13*	9.41 ± 0.16*	8.30 ± 0.10*
ZBSE	250	3.74 ± 0.05*	4.56 ± 0.05*	5.90 ± 0.09*	5.05 ± 0.05*	4.50 ± 0.15*
	500	3.82 ± 0.04*	5.49 ± 0.09*	6.61 ± 0.07*	6.02 ± 0.16*	5.00 ± 0.16*

Values are expressed as mean ± standard error for mean, *n*: number of mice (6); *P < 0.001 versus control (Student's *t*-test).
